# State transitions in *Physcomitrium patens* studied with time-resolved fluorescence

**DOI:** 10.1007/s11120-026-01206-4

**Published:** 2026-03-06

**Authors:** Dana Verhoeven, Cleo Bagchus, Lotte Jore, Herbert van Amerongen, Emilie Wientjes

**Affiliations:** Laboratory of Biophysics, Wageningen University, Wageningen, 6708 WE The Netherlands

**Keywords:** Photosynthesis, Light harvesting, Time-resolved fluorescence, Photosystem I, *Physcomitrium patens*, State transitions

## Abstract

Photosynthetic organisms adapt their light-harvesting apparatus continuously to the ever-changing light environment. The model moss *Physcomitrium patens* shares acclimation responses with both the vascular plant *Arabidopsis thaliana* and the green alga *Chlamydomonas reinhardtii*. In vivo time-resolved fluorescence spectroscopy has previously helped to clarify the mechanisms of acclimation in *A. thaliana* and *C. reinhardtii*. However, it has not yet been applied to study acclimation responses in *P. patens*. In this work, we studied state transitions in *P. patens* with in vivo time-resolved fluorescence spectroscopy. We found one short (35–45 ps) and one longer (165–190 ps) in vivo photosystem I (PSI)-related fluorescence lifetime. We suggest that the short fluorescence lifetime is associated with PSI with a small, plant-like antenna, whereas the second component arises from PSI with a larger antenna size. The PSI antenna size is affected by state transitions, a dynamic process that balances the energy capture between PSI and photosystem II (PSII). We found that state transitions in *P. patens* act most strongly at an exceptionally low light intensity of 3 µmol m^− 2^ s^− 1^ actinic light. The in vivo time-resolved fluorescence spectra suggest that state transitions at this actinic light intensity mainly affect PSII and PSI-large, despite PSI-large being excited far less than PSI-small. Our findings indicate that state transitions in *P. patens* rebalance the excitation-energy distribution to a similar level as vascular plants, but at different light intensities. Our results suggest that state 1 to state 2 transitions primarily increase the abundance of PSI-large.

**Supplementary Information:**

The online version contains supplementary material available at 10.1007/s11120-026-01206-4.

## Introduction

Light absorption drives charge separation in photosystem I (PSI) and photosystem II (PSII). Photosynthetic organisms ideally use all absorbed energy for photochemistry when the light intensity is low. However, when the light intensity is high, they need to protect themselves against photodamage. Since light quality and quantity vary over time from seconds to seasons (Demmig-Adams et al. 2012; Kaiser et al. 2018; Ruban 2009; Wang et al. 2020), dynamic acclimation responses are essential for plant fitness and yield production (Frenkel et al. 2007; Karim 2021). When the light intensity is low, state transitions maximize light harvesting by shuttling part of the antenna between PSI and PSII, as such optimizing the excitation pressure on both photosystems (Allen 1992). When the light intensity is high, non-photochemical quenching (NPQ) dissipates excess absorbed energy as heat (Horton et al. 1996; Li et al. 2000; Niyogi et al. 1998). Both acclimation responses affect the fluorescence properties of either one or both of the photosystems. For example, NPQ shortens the PSII lifetime with closed reaction centres (Farooq et al. 2018; Holzwarth et al. 2009), whereas state transitions alter the respective contributions of PSI and PSII fluorescence emission (Bhatti et al. 2020; Bos et al. 2019; Iwai et al. 2010; Ünlü et al. 2014; Verhoeven et al. 2023).

PSI and PSII contain a core complex with a reaction centre to allow charge separation and an outer antenna system to increase the absorption cross section. The fluorescence emission of PSI and PSII can be separated with time-resolved fluorescence spectroscopy, even though their emission spectra overlap (Akimoto et al. 2014; Holzwarth et al. 2009; Wientjes et al. 2013a). This separation is based on the short fluorescence lifetime of PSI (50–100 ps in vascular plants) and the long fluorescence lifetime of PSII with closed reaction centres (1500–2000 ps in vascular plants) (Belgio et al. 2012; P. R. Bos et al. 2023; Ihalainen et al. 2005; Wientjes et al. 2011, 2017). Excited-state kinetics provide insight into how efficiently absorbed light energy is transferred and trapped by the photosystems. By resolving fluorescence lifetimes, we can assess light-harvesting efficiency and monitor dynamic changes in energy transfer pathways. This approach can also be used for studying how the photosynthetic apparatus acclimates to changing light conditions, for example during state transitions or NPQ. Here, we investigate the model moss *Physcomitrium patens* (*P. patens*), which possesses 2 types of PSI-complexes: PSI-LHCI and PSI-megacomplex (Iwai et al. 2015). The PSI-LHCI complex is structurally similar to PSI in vascular plants. It has a PSI core complex and a light harvesting complex I (LHCI) belt, composed of 4 Lhca pigment-protein complexes (Gorski et al. 2022). The PSI-megacomplex consists of PSI-LHCI with additional, moss-specific, Lhcb9, light harvesting complex II (LHCII) and an extra LHCI belt (Sun et al. 2023; Zhang et al. 2023). The PSI-megacomplex is only formed when Lhcb9 is expressed (Iwai et al. 2015; Pinnola et al. 2018). Lhcb9 expression is enhanced by growing the moss on medium with glucose in low-light conditions (17 µmol m^− 2^ s^− 1^) (Pinnola et al. 2018). The fluorescence decay kinetics of PSI and PSII in *P. patens* have not been studied until recently. Liu et al. reported 46 ps as the in vitro fluorescence lifetime of PSI-small (Liu et al. 2025). However, we do not know the in vivo time-resolved fluorescence lifetimes and spectra of *P. patens* yet.

In vivo time-resolved fluorescence spectroscopy has been used to study state transitions in both plants and green algae (Bos et al. 2019; Iwai et al. 2010; Verhoeven et al. 2023). State transitions improve photosynthetic efficiency by rebalancing the excitation pressure on PSI and PSII (Minagawa 2011; Ruban and Johnson 2009; Taylor et al. 2019). Since the absorption spectra of PSI and PSII differ, changes in the light spectrum can disturb their excitation balance. However, this excitation pressure is preferentially balanced for optimal light-harvesting efficiency, because PSI and PSII work in series in the linear electron transport chain (Pfannschmidt 2005; Taylor et al. 2019; Walters 2005). PSI absorbs more far-red and green light, while PSII absorbs more blue light (Hogewoning et al. 2012; Johnson and Wientjes 2020; Mattila et al. 2020). When PSI is overexcited compared to PSII, state 1 is induced, where LHCII mostly transfers excitation energy to PSII. Instead, when PSII is overexcited, the plastoquinone-pool (PQ-pool) becomes reduced and activates the Stn7 protein kinase. Stn7 phosphorylates the mobile part of the LHCII pool that is associated with PSII. Upon phosphorylation, LHCII migrates to PSI and transfers energy to PSI (Allen et al. 1981; Bellaflore et al. 2005). The protein phosphatase TAP38/PPH1 facilitates the reverse process by dephosphorylating LHCII (Pribil et al. 2010; Shapiguzov et al. 2010). As a consequence, state transitions alter the ratio of PSI and PSII absorption and emission. Several mechanisms are suggested for this effect in green algae. On the one hand, the shuttling of LHCII complexes between photosystems could alter the PSI and PSII fluorescence emission (Nawrocki et al. 2016). On the other hand, LHCII could disconnect from PSII, but not associate with PSI. Instead, disconnected LHCII could aggregate and quench, resulting in a fluorescence lifetime component of several hundreds of picoseconds (Iwai et al. 2010; Ünlü et al. 2014). Previous studies on cyanobacteria and green algae illustrate that state transitions do not always enlarge the antenna system of PSI (Choubeh et al. 2018; Le Quiniou et al. 2015; Ünlü et al. 2014).

Although the mechanism of state transitions is extensively studied in vascular plants and green algae, we do not know yet how it works in *P. patens*. *P. patens* is an interesting model organism, because it is a descendant of an evolutionary intermediate between green algae and vascular plants (Iwai and Yokono 2017; Rensing et al. 2018, 2020). Therefore, its acclimation responses resemble both those of vascular plants and green algae. For example, NPQ in *P. patens* is regulated by zeaxanthin, PsbS (vascular plants) and LHCSR (green algae) (Alboresi et al. 2010; Furukawa et al. 2019; Gerotto et al. 2011; Pinnola et al. 2015). Hence, the knowledge on state transitions in plants and green algae is probably valuable to understand the mechanism in *P. patens*. The qT parameter quantifies state transitions by monitoring the PSII antenna decrease when the photosynthetic machinery adapts from state 1 to state 2 (Haldrup et al. 2001). In the plant *A. thaliana* the qT value ranges from 8 to 13% (Benson et al. 2015; Bressan et al. 2018; Damkjær et al. 2009; Koskela et al. 2018; Wientjes et al. 2013b), while in the algae *C. reinhardtii*, qT was estimated up to values as high as 80% (Delosme et al. 1996). However, more recently, the *C. reinhardtii* PSI absorption cross section was estimated to increase to a lesser extent, ~ 5%-35% upon state 1 to 2 transition (Nawrocki et al. 2016; Ünlü et al. 2014, 2016). In *P. patens*, qT values ranging from 1 to 13% were reported. Often those reports do not specify the actinic light intensity used to measure these values (Gerotto et al. 2022; Iwai et al. 2015; Pinnola et al. 2018). When specified, the light intensity is close to the growth light intensity (Busch et al. 2013), while in *Arabidopsis thaliana* (*A. thaliana*) usually 10–30% of the growth light intensity is used to avoid activation of NPQ (Benson et al. 2015; Haldrup et al. 2001; Koskela et al. 2018; Nikkanen et al. 2019; Pribil et al. 2010). Nonetheless, multiple studies agree that the presence of Lhcb9 does not influence the qT parameter, suggesting that the PSI-megacomplex is not involved in state transitions (Iwai et al. 2015; Pinnola et al. 2018).

In this study, we use ultrafast streak-camera measurements to gain insight into the kinetics of in vivo PSI excitation energy trapping in *P. patens*. We promote Lhcb9 expression with glucose in the growth medium and low growth light intensity (17 µmol m^− 2^ s^− 1^) to see the effect of extra PSI-megacomplex formation on the spectra. Furthermore, we evaluate state transitions at different actinic light intensities by using pulsed-amplitude modulation (PAM). Finally, we apply the outcome of these measurements to compare in vivo time-resolved fluorescence spectra of *P. patens* in state 1 and state 2. Resolving these spectra under state transitions sheds light on how early land plants regulate energy distribution between photosystems.

## Materials and methods

### Plant material

*Physcomitrium patens* Gransden strain WT and *stn7* KO (Gerotto et al. 2022) were grown on minimal medium (Ashton et al. 1979) supplemented with 0.5 µM ammonium tartrate. The mosses grew in a growth chamber under 50 µmol m^− 2^ s^− 1^ white light with a photoperiod of 16 h and 60% relative humidity. In experiments with glucose treatment (Fig. 2), the growth medium was supplemented with 0.5% glucose and the light intensity was lowered to 17 µmol m^− 2^ s^− 1^. All experiments were performed on protonemal tissue between 5 and 15 days of age.

### PAM measurements

We measured chlorophyll (Chl) fluorescence while inducing state transitions with the Walz Mini-PAM II (Heinz Walz GmbH, Effeltrich Germany). The measurement sequence was adapted from the state transition measurement of Haldrup et al. (Haldrup et al. 2001). Before the start of the measurements, the moss was dark adapted overnight. At the start of the sequence, the optical fiber was placed at minimal distance of the protonemal tissue and F_v_/F_m_ was measured. Next, the mosses were exposed to three light phases, each ending with a saturating light pulse of 6000 µmol m^− 2^ s^− 1^ for 1 second. In the first light phase, the mosses were exposed to red actinic and far-red light for 10 minutes, after which the variable F_m_’ was measured with the saturating pulse. In the second light phase, the mosses were exposed to only actinic light for 20 min, facilitating the transition to state 2. In the third light phase, the mosses were exposed to actinic and far-red light for 20 min, facilitating the transition back to state 1. At the beginning of this light phase, the variable F_m2_’ was measured with a saturating pulse. At the end, the variable F_m1_’was measured with a saturating pulse. Between different measurements, we varied the actinic light intensity between 3 µmol m^− 2^ s^− 1^, 10 µmol m^− 2^ s^− 1^ and 52 µmol m^− 2^ s^− 1^. We calculated the qT and NPQ from the values obtained with the saturating pulses following Eq. 1 (Ruban and Johnson 2009) and 2.

1 $$qT=\frac{F{m}_{1}^{{\prime}}-F{m}_{2}^{{\prime}}}{F{m}_{1}^{{\prime}}}\times100\%$$

2 $$NPQ=\frac{Fm}{Fm{\prime}}-1$$

### Blue native PAGE gel

We performed Blue Native PAGE gel electrophoresis to confirm the presence of the state transition complex in blue light treated WT plates. Three WT and three *stn7* plates were dark adapted overnight. Then the protonemata were exposed for 30 min to either darkness, 3 µmol m^− 2^ s^− 1^ blue light (λ_max_ = 488 nm) or 3 µmol m^− 2^ s^− 1^ far-red light (λ_max_ = 706 nm). Afterwards, the protonemata were harvested and thylakoids were isolated based on the protocol of Caffarri et al. (Caffarri et al. 2009). The material was kept on ice and in the dark during the isolation. Protonemata were homogenized in buffer 1 (400 mM sorbitol, 5 mM EDTA, 10 mM NaHCO3, 5 mM MgCl_2_, 20 mM tricine and 10 mM NaF) with a homogenizer (Homogenizer 150, Fischerbrand). The suspension was filtered through a 100 µM mesh filter and centrifuged for 4 min at 4000 g. The pellet was resuspended in buffer 2 (300 mM sorbitol, 2.5 mM EDTA, 5 mM Mgcl2, 20 mM tricine, 10 mM NaHCO3, 10 mM NaF) and centrifuged for 4 min at 4000 g. Then the pellet was resuspended in buffer 3 (5 mM MgCl2, 2.5 mM EDTA, 20 mM HEPES and 10 mM NaF) and centrifuged for 10 min at 10 000 g. The resulting pellet was resuspended in buffer 1 and stored at -20 °C.

In the next steps, the thylakoids were solubilized and loaded onto a gel. Also, during this procedure, the materials were kept on ice. The prepared thylakoids (40 µg) were defrosted on ice and washed twice with wash buffer (330 mM sorbitol, 50 mM Bis-Tris (pH 7)). After each washing step the suspension was centrifuged for 5 min at 5000 g. The resulting pellet was resuspended in 40 µL resuspension buffer (25 mM Bis-Tris (pH 7), 20% glycerol) and 40 µL solubilization buffer (25 mM Bis-Tris (pH 7), 20% glycerol, 2% n-dodecyl- β-D-maltoside (β-DM)). This thylakoid solution was solubilized with a final concentration of 1% β-DM on a rocking platform on ice for 30 min. Then the solution was centrifuged at 18,000 *g* for 20 min to remove insoluble material. Per sample, 15 µg of Chl from the supernatant was loaded onto a pre-cast Blue Native Page gel (NativePAGE™ 3–12% Bis-Tris gel, Invitrogen). The gel ran at 4 °C with anode buffer (5 mM Bis-Tris) and cathode buffer (50 mM tricine, 15 mM Bis-Tris (pH 7), 0.02% CBB G-250). After the samples migrated through 1/3 of the gel, the blue cathode buffer was switched for a clear cathode buffer without Coomassie staining. The gel ran at 75 V for 45 min, 150 V for 3 h and then 200 V for approximately 3 h until the dye came off the gel.

### Sucrose gradient

Thylakoid isolation of dark adapted WT protonemata was done as described above. The fresh thylakoids were diluted with buffer 5 (20 mM Tricine-KOH pH 7.8, 0.1 M EDTA) to a Chl concentration of 0.5 mg/mL. Next, 10% (w/v) β-DM was added to a final Chl concentration of 0.5 mg/mL and a final β-DM concentration of 1% (w/v). This thylakoid suspension was solubilized on ice for 30 min. Then, the suspension was centrifuged (15000 g for 10 min at 4 °C) and the supernatant was loaded on a sucrose gradient (0.1–1.3 M sucrose, 25 mM MES-NaOH pH 6.5, 0.03% β-DM). The sucrose gradients were centrifuged with rotor SW41Ti at 40 000 rpm for 18 h at 4 °C. The bands were collected with a syringe and kept on ice.

### In vivo time-resolved fluorescence

We recorded in vivo time-resolved fluorescence spectra of *P. patens* protonemata with a streak camera (C10910, Hamamatsu) setup (Bos et al. 2019; Farooq et al. 2018; van Stokkum et al. 2008). The mosses were dark adapted for 1 h prior to the measurement. The full plate with protonemal tissue was placed inside a rotating cell, rotating at 3 rpm and moving sideways with 1 rpm. The tissue was excited with a pulsed white light laser (Rock white light laser, Leukos) at wavelength 488 ± 10 nm. The laser has a repetition rate of 38 MHz, pulse width of 6 ps, 40 µW laser power and a spot size of approximately 200 μm. The Chl fluorescence above 640 nm is spectrally separated with a grating of 150 g/mm (Shamrock, Andor). The fluorescence was recorded with 1 s integration time over 600 integrations, in a time window of approximately 2 ns. This creates a streak image where the fluorescence photon counts are separated by wavelength and arrival time at the detector. For measurements with DCMU (3-(3,4-dichlorophenyl)-1,1-dimethylurea), protonemata were incubated with DCMU buffer (30 µM DCMU, 150 mM sorbitol and 10 mM Hepes) 1 h prior to the measurement. For measurements without DCMU, we aimed to close the reaction centres with an additional non-pulsed laser (λ = 532 nm, intensity 0.5 mW and spot size of approximately 1 mm) focused on the same spot as the pulsed laser. DCMU was not used in state transition measurements, because it influences the state transitions. In the state transition measurements, the same WT and s*tn7* protonemata plates were measured after exposure to three different light conditions. Firstly, we recorded a streak image of the dark-adapted protonemata. Secondly, these plates were exposed to 3 µmol m^− 2^ s^− 1^ blue light (λ_max_ = 488 nm) for 15 min to transition into state 2 and an image was recorded with the blue light still on. Lastly, the protonemata were adapted to 3 µmol m^− 2^ s^− 1^ far-red light (λ_max_ = 706 nm) for 45 min and an image was recorded afterwards.

The spectra resulting from these measurements were corrected for background signal and wavelength-dependent sensitivity of the detector. The data was analysed with R-package TIMP based software Glotaran (Snellenburg et al. 2012). The analysis resulted in decay associated spectra (DAS) showing an emission spectrum for every fluorescence lifetime component in the streak image. For normalization, the total summed area of the 3 DAS from 665 nm to 780 nm is set to 1.

## Results

**Fig. 1:**
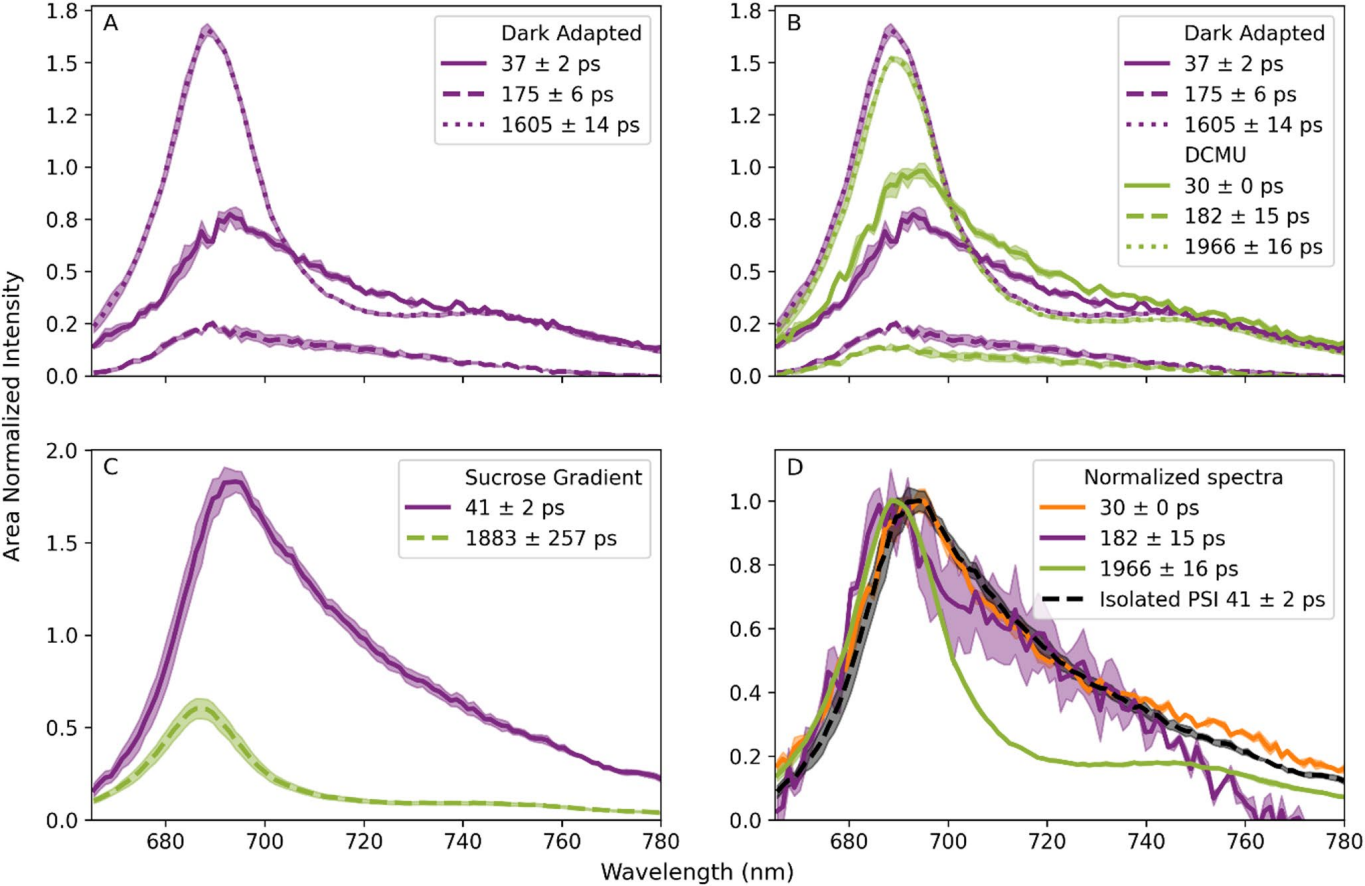
Decay associated spectra (DAS) of P. patens obtained with in vivo time-resolved fluorescence spectroscopy. **A**) WT in dark-adapted state, excitation wavelength 488 nm (*n* = 3), **B**) WT in dark adapted state (same as A) and WT infiltrated with DCMU to close all PSII reaction centres, excitation wavelength 488 nm, **C**), Isolated PSI-small rich fraction from a sucrose gradient (*n* = 3), excitation wavelength 488 nm, **D**), Normalized decay-associated spectra (DAS) of WT protonemata incubated with DCMU. In addition, the DAS of the isolated PSI complex from Fig. 1C is added. The shaded area shows the SE. To compare DAS from different conditions (**B**) the sum of the 3 DAS is normalized to the same area

In this work we recorded in vivo time- and wavelength-resolved fluorescence data to gain insight into the in vivo energy trapping kinetics of PSI in *P. patens*. To clearly separate the kinetics of PSI from PSII we aimed to close the PSII reaction centres with laser light to increase their fluorescence lifetime (see M&M). The analysis resulted in decay associated spectra (DAS), each corresponding to the emission spectrum of a fluorescence lifetime component in the recorded image. The dark-adapted WT protonemata showed three DAS with respective lifetimes around 40 ps, 180 ps and 1600–1900 ps (Fig. 1A). The longest lifetime component (1600–1900 ps) matches the spectrum and lifetime of dark adapted PSII with closed reaction centres in *A. thaliana* (Fig. 1D) (Wientjes et al. 2017). The shorter lifetime components (37 ps and 175 ps) are likely related to PSI, but might also have contributions from PSII (Fig. 1A and D).

To assign these components more confidently to PSI or PSII, we performed additional experiments. Firstly, open PSII reaction centres of plants have a fluorescence lifetime of 170–330 ps and could therefore interfere with the 175 ps component (Keuper and Sauer 1989; Van Oort et al. 2010; Wientjes et al. 2013b). Hence, WT protonemata were incubated with DCMU (3-(3,4-dichlorophenyl)-1,1-dimethylurea) to chemically close all PSII reaction centres in presence of laser light. The PSII fluorescence lifetime was ~ 1900 ps in presence of DCMU and ~ 1600 ps without, indicating that the reaction centers were not fully closed in absence of DCMU (Fig. 1B). The DAS of DCMU treated protonemata resemble the DAS of dark-adapted protonemata, even though their respective contributions and fluorescence lifetimes slightly differ (Fig. 1B). This means that open PSII reaction centres can only partially explain the 175 ps lifetime component. On the other hand, closed PSII reaction centre core complexes from cyanobacteria exhibit multiexponential decay kinetics with a lifetime component around 200 ps (Szczepaniak et al. 2009). Therefore, we cannot exclude a contribution from closed PSII reaction centres to the measured ~ 175 ps component. However, this contribution is unlikely to fully explain this component, since its amplitude decreases when the reaction centres are chemically closed with DCMU (Fig. 1B). In addition, the spectrum of the ~ 200 ps closed reaction center component in cyanobacterial PSII cores closely matches that of the 1500–2000 ps PSII component (Tian et al. 2013), rather than the spectrum of the ~ 175 ps *P.patens* component measured here (Fig. 1B, D). Secondly, we isolated PSI-LHCI on a sucrose gradient and recorded time-resolved fluorescence spectra (Fig. 1C). We found a fluorescence lifetime of 41 ps, in close agreement with the isolated PSI-LHCI average lifetime of 46 ps found by (Liu et al. 2025). The 1883 ps component is attributed to contamination with PSII (Fig. 1C). Furthermore, the isolated PSI spectrum resembles the spectrum of the 30 ps component of WT *P.patens* protonemata incubated with DCMU (Fig. 1D). Notably, the emission maximum of the ~ 175 ps component appears slightly blue-shifted compared to the ~ 40 ps component, even though the general shape of their spectra is similar (Fig. 1D). These experiments indicate that the ~ 40 ps and ~ 175 ps components both have a PSI origin.

**Fig. 2:**
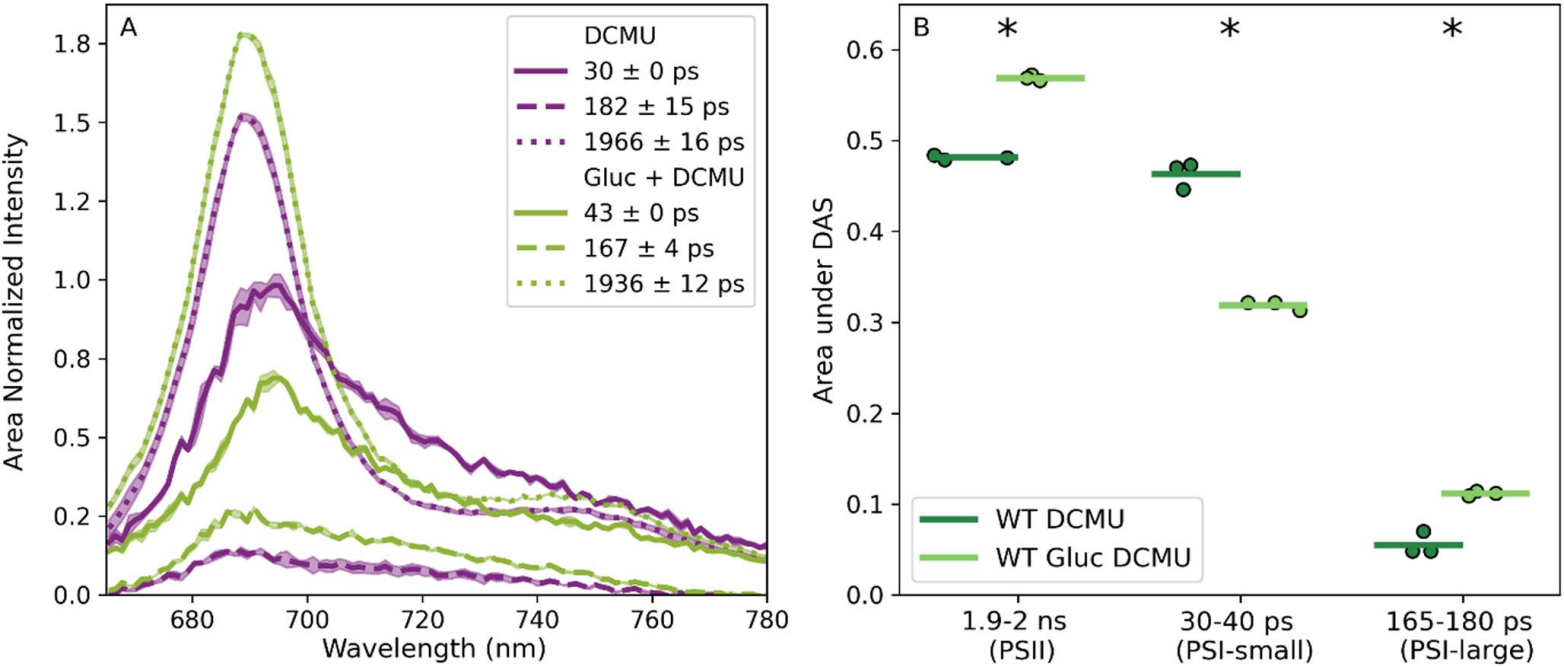
**A**) Decay associated spectra (DAS) of WT infiltrated with DCMU grown on minimal medium in 50 µmol m^− 2^ s^− 1^ or on minimal medium supplemented with 0.5% glucose in 17 µmol m^− 2^ s^− 1^ (*n* = 3). **B**) Area under the DAS in A) for every fluorescence lifetime (*n* = 3), excitation wavelength 488 nm. The assignment of photosystems was done based on the fluorescence lifetimes. The stars (*) indicate statistical significance based on a one-way ANOVA followed by a Tukey post hoc test (*p* < 0.05)

The fluorescence lifetime scales with the antenna size, if the light-harvesting antenna are well-connected (Caffarri et al. 2011). Hence, the long PSI trapping time of the ~ 175 ps component suggests that the PSI antenna size is substantially larger and/ or that antenna are poorly connected leading to slow transfer and trapping of the excitation energy. We hypothesized that this component could arise from the Lhcb9 dependent PSI megacomplex. To alter the balance between PSI with a small and large antenna size we grew WT *P. patens* on glucose-enriched medium at a lower light intensity (17 µmol m^− 2^ s^− 1^) compared to standard conditions (minimal medium, 50 µmol m^− 2^ s^− 1^ light). Under such conditions, increased Lhcb9 expression has previously been reported(Pinnola et al. 2018). We note that Lhcb9 expression levels were not quantified in the present study. In these glucose-grown protonemata, the relative amplitude of the long fluorescence lifetime components (~ 175 ps and ~ 1.9 ns) increased, while the relative amplitude of the short lifetime component (30–40 ps) decreased (Fig. 2). This indicates a shift in the trapping kinetics associated with changes in photosystem organization or antenna composition. The protonemata were infiltrated with DCMU, hence the shift in trapping kinetics cannot be explained by open PSII reaction centres. As the amplitude of the ~ 175 ps component increases in presence of glucose, this hints to the involvement of the PSI megacomplex. Alternatively, excitation-energy transfer from LHCII that is not part of a specific PSI megacomplex can also lead to such a fluorescence lifetime. LHCII is abundant in the stroma lamellae of *P. patens* (Pinnola et al. 2015) and association of LHCII with PSI increases its fluorescence lifetime in *A. thaliana* and *C. reinhardtii* (Le Quiniou et al. 2015; Schiphorst et al. 2022). To sum up, these results indicate that the in vivo fluorescence lifetime of PSI-small is 35–45 ps, PSI-large is 165–190 ps and PSII with closed reaction centers is 1600–1950 ps.

**Fig. 3:**
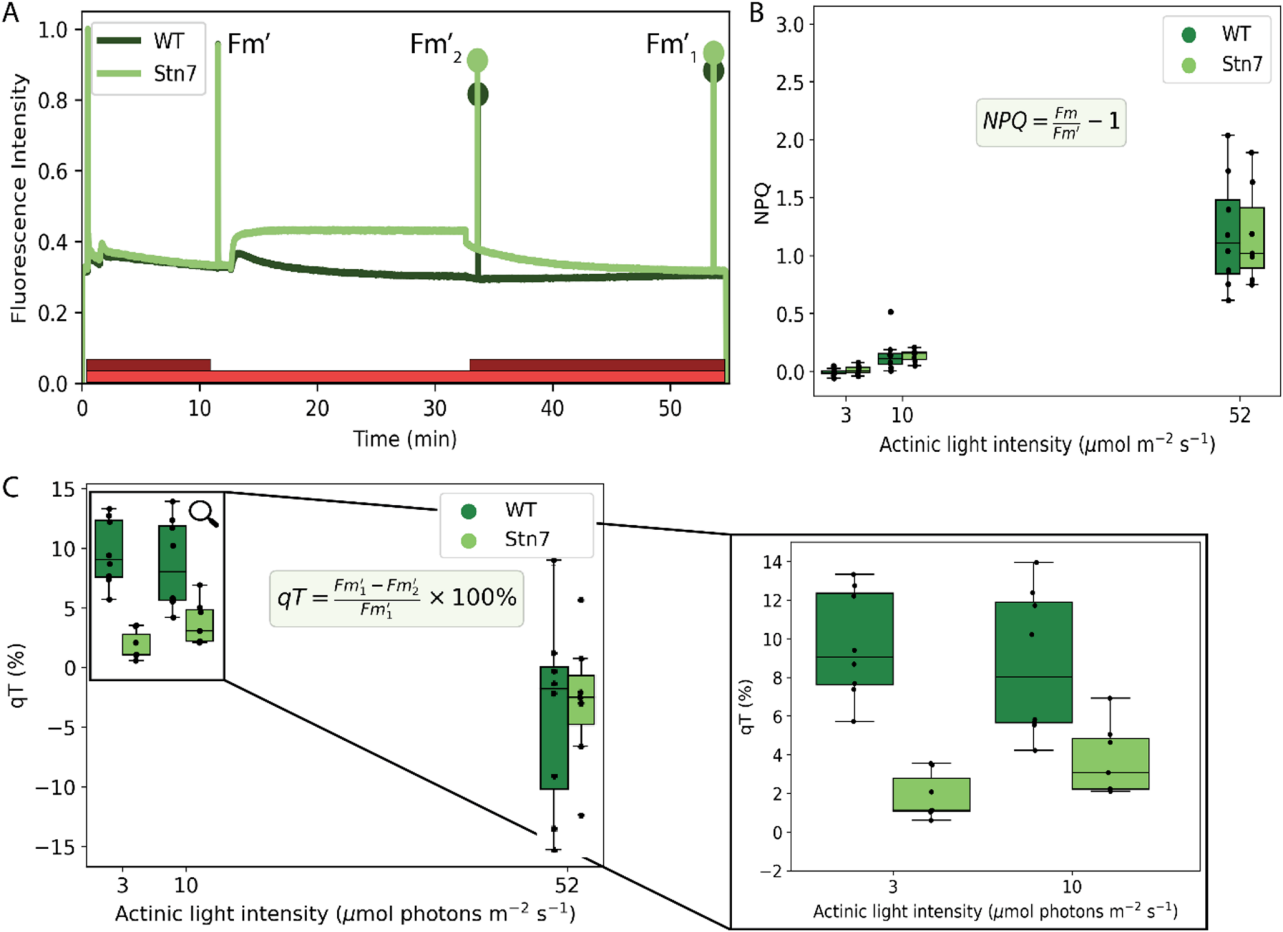
**A**) An example of a PAM state transition measurement in WT and stn7. The red bar indicates when the actinic light (3 µmol m^-2^ s^-1^ in this example) was on, and the dark-red bar indicates when the far-red light was on. The dots indicate the fluorescence intensity after a saturating pulse in state 1 (F_m1_) and state 2 (F_m2_). PAM measurements like in this example are used for calculating the parameters plotted in **B**) and **C**). **B**) NPQ value of WT and stn7 measured at various actinic light intensities (*n* = 8). NPQ values are calculated using the Stern-Volmer equation in the figure. **C**) qT value of WT and stn7 measured at various actinic light intensities (*n* = 8)

To investigate in vivo excitation energy trapping in state 1 and state 2, we recorded both state transition-related parameters (qT) and non-photochemical quenching (NPQ) with PAM fluorometry. We monitored NPQ, because *P. patens* develops substantially stronger NPQ than vascular plants, even at low light intensities. This could interfere with accurate determination of qT. During the measurement, the actinic red light was continuously on, and the far-red light was switched on and off to either overexcite PSI or PSII (Fig. 3A). In the WT, the F’ signal sharply increased when the far-red light was switched off and decreased over time when the moss acclimated to state 2. In the s*tn7* mutant, the F’ signal increased after switching off the actinic light but did not decrease over time (Fig. 3A). These PAM traces were recorded with 3, 10 and 52 µmol m^− 2^ s^− 1^ actinic light intensity, yielding F_m_ (dark adapted), F_m_’ (after 10 min of acclimation to actinic and far-red light), F_m2_^’^ (state 2) and F_m1_^’^ (state 1). In the first measurement phase, the actinic light and the far-red light were both on to keep the plants in state 1 and investigate the NPQ effect of the actinic light (Fig. 3B). NPQ increased with increasing light intensity in both genotypes (Fig. 3B). At 52 µmol m^− 2^ s^− 1^ we measured an NPQ value of 1.0 on average, in agreement with (Gerotto et al. 2022). The qT parameter, indicating the change of PSII antenna size during the state 1 to state 2 transition, reached 9% on average in WT with 3 µmol m^− 2^ s^− 1^ actinic light intensity (Fig. 3C). In *stn7*, qT was expected to be 0% because state transitions are supposed to be absent, but at 52 µmol m^− 2^ s^− 1^ both genotypes showed a large spread in qT values with an average value around − 1% (Fig. 3C). This variation coincided with higher and more variable NPQ (Fig. 3B), suggesting that strong qE-type quenching under high light interferes with reliable qT determination.

**Fig. 4:**
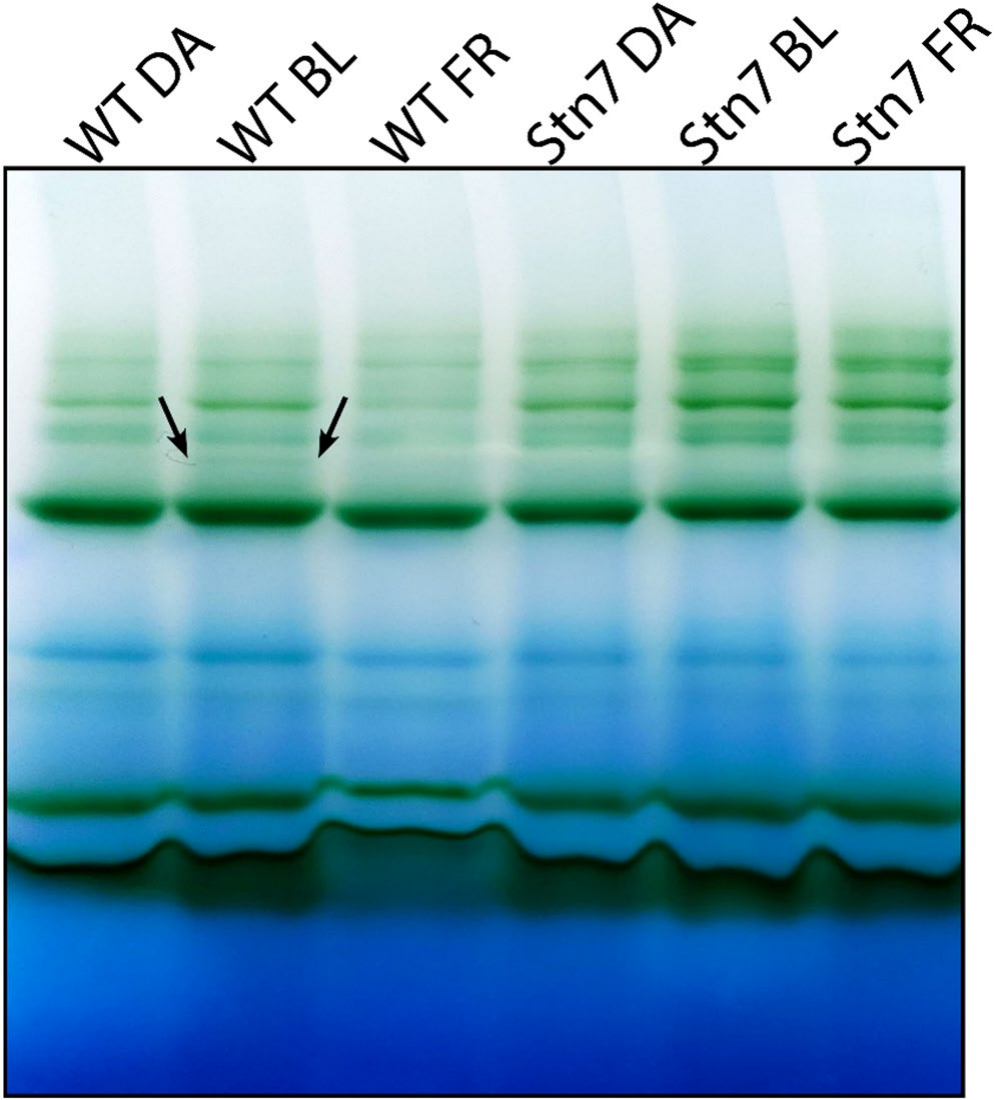
Blue-Native PAGE gel of WT and stn7 thylakoids. The protonemata were dark adapted overnight and treated with either no light, 3 µmol m^-2^ s^-1^ blue light (BL) or 3 µmol m^-2^ s^-1^ far-red light (FR) for 30 min prior to the thylakoid isolation. The isolated thylakoids were dissolved with 1% β-DM. The arrow points towards a band that is supposed to be the PSI(core)-LHCI-LHCII state 2 complex, based on 2D-SDS-BN-PAGE gels showing absence of Lhcb9 in this band (Gerotto et al. 2022)

We confirmed that low light intensity induces state transitions with Blue-Native PAGE gel electrophoresis on WT and *stn7* protonemata acclimated to darkness, or 3 µmol m^− 2^ s^− 1^ blue or far-red light (Fig. 4, full gel in SI Fig. 2). The thylakoids were isolated from the protonemata and solubilized with 1% β-DM (n-dodecyl- β-D-maltoside). After solubilization, the membrane supercomplexes were loaded onto a Blue-Native PAGE gel. The lane of WT treated with blue light shows a faint band that does not appear in other lanes, indicated with an arrow (Fig. 4). This band represents the state-2 complex (PSI-LHCI-LHCII) (Gerotto et al. 2022). Following these findings, we induced state transitions with 3 µmol m^− 2^ s^− 1^ blue light in the in vivo time-resolved fluorescence setup.

**Fig. 5:**
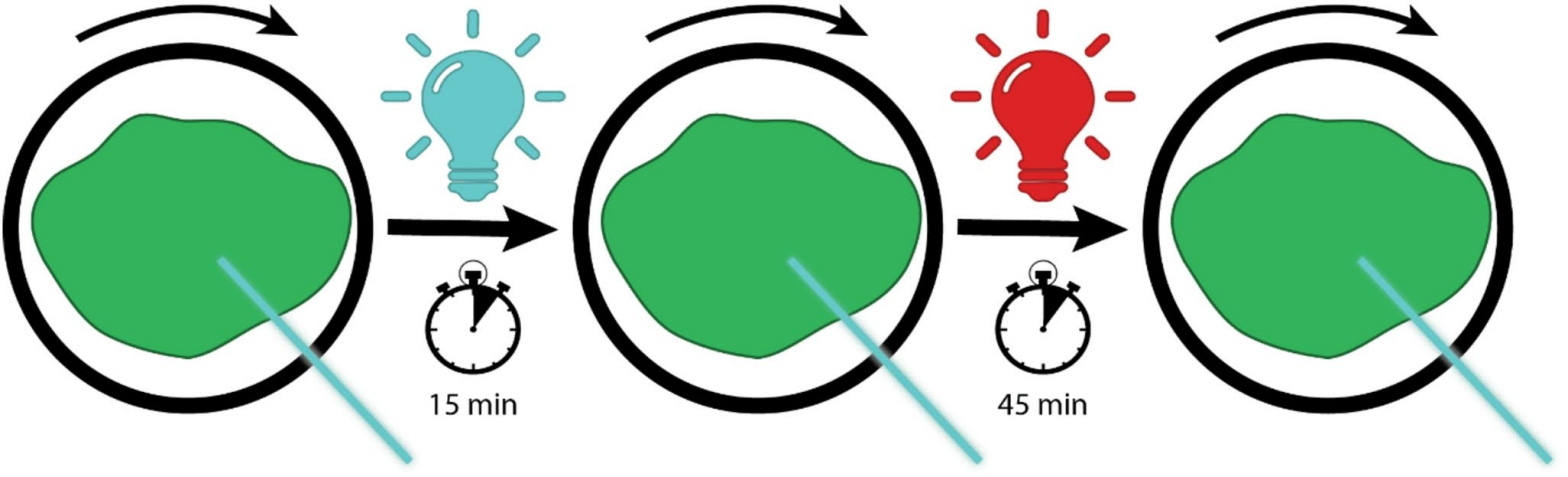
Schematic drawing of in vivo time-resolved fluorescence measurements to study state transitions. Protonemata (grown on minimal medium) are first measured in a dark-adapted state (state 1). Then an in vivo time-resolved fluorescence spectrum is measured after 15 min of acclimation to 3 µmol m^-2^ s^-1^ blue light (state 2) and again after 45 min of illumination with far-red light (state 1)

**Fig. 6:**
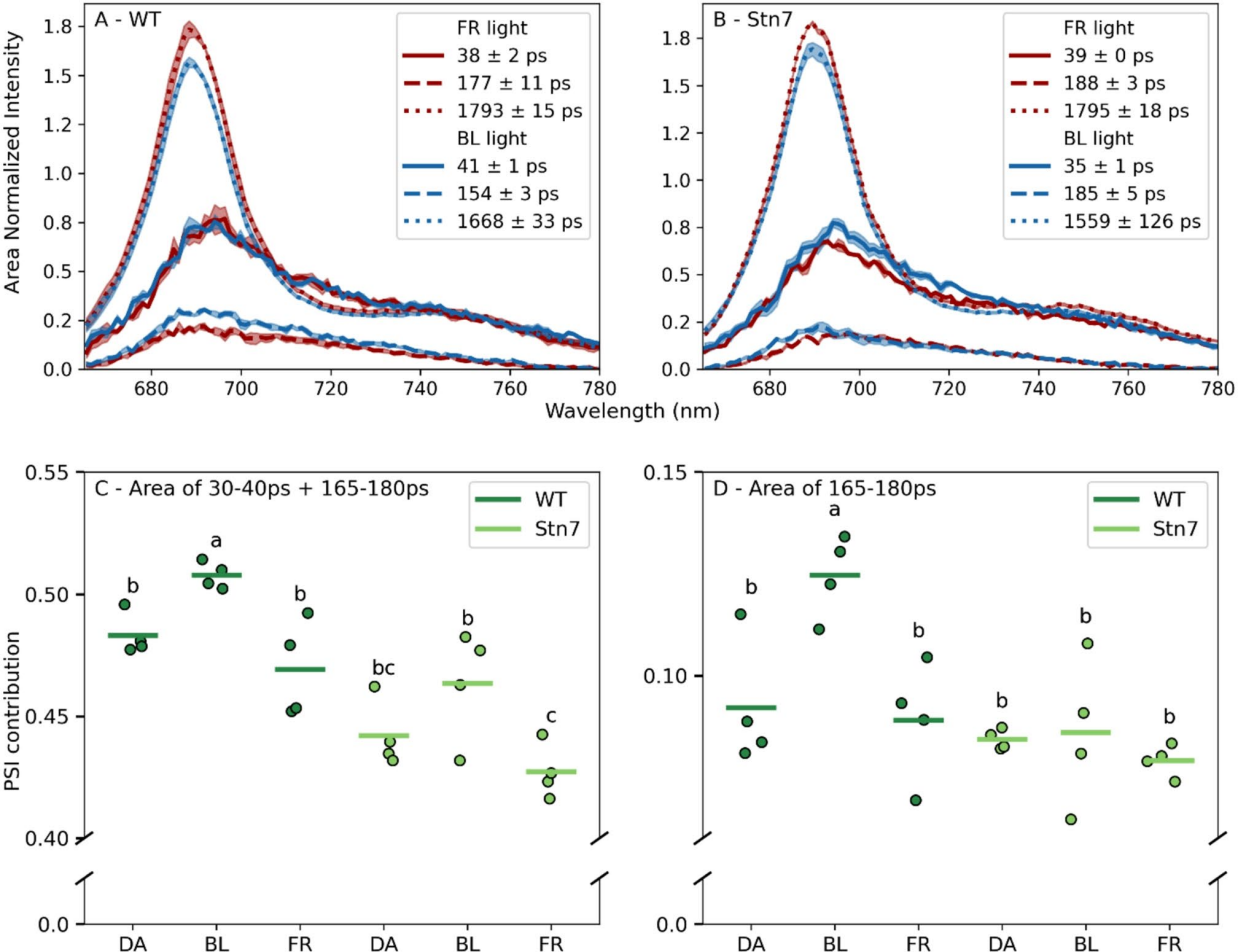
**A**) Decay associated spectra (DAS) of WT obtained with in vivo time-resolved fluorescence spectroscopy after acclimation to 3 µmol m^-2^ s^-1^ blue or 3 µmol m^-2^ s^-1^ far-red light (*n* = 4). **B**) Decay associated spectra (DAS) of stn7 obtained with in vivo time-resolved fluorescence spectroscopy after acclimation to 3 µmol m^-2^ s^-1^ blue or 3 µmol m^-2^ s^-1^ far-red light (*n* = 4), excitation wavelength 488 nm. The shaded area shows the SE. **C**) Total PSI contribution of WT and stn7 after dark adaptation (DA) or adaptation to blue (BL) or far-red (FR) light. The sum under the 3 DASs is normalized to 1. The total PSI contribution is the sum of the area under the spectrum of the 35–40 ps component and the 170–190 ps component. The letters indicate statistical significance based on a one-way ANOVA followed by a Tukey post hoc test **D**) PSI-large contribution of WT and stn7 after dark adaptation (DA) or adaptation to blue (BL) or far-red (FR) light. The PSI-large contribution is the area under the spectrum of the 170–190 ps component. The letters indicate statistical significance based on a one-way ANOVA followed by a Tukey post hoc test (*p* < 0.05)

We measured the in vivo time-resolved fluorescence spectra of WT and *stn7* protonemata in the order: dark adapted, state 2-conditions (3 µmol m^− 2^ s^− 1^ blue light) and state 1 conditions (3 µmol m^− 2^ s^− 1^ far-red light). This is schematically visualized in Fig. 5. The amplitudes of the PSI- and PSII-associated DAS are determined by their relative excitation with the laser light. Consequently, changes in these amplitudes reflect a redistribution of excitation energy of LHCII from PSII to PSI during the state 1 to state 2 transition. The external lamps were verified to induce state transitions by PAM fluorometry similar to Fig. 3A (SI Fig. 3). In WT, the PSII contribution decreased, and the PSI-large contribution increased upon the transition from state 1 to state 2 (Fig. 6A). The light treatments did not affect the PSI-small contribution (Fig. 6A). In s*tn7*, the PSII contribution seemed to decrease slightly, and the PSI-small contribution increased slightly in state 2 conditions compared to state 1 conditions (Fig. 6B). Quantifying the area under the different spectra showed that the WT protonemata treated with blue light have the largest summed PSI contribution (Fig. 6C). For *stn7*, the summed PSI contribution did not differ significantly between the three light treatments. Regarding just the contribution of PSI large, it is significantly larger in WT protonemata treated with blue light compared to all other samples (Fig. 6D).

## Discussion

### Two PSI-related fluorescence lifetime components

In this work, we recorded in vivo time-resolved fluorescence spectra of *P. patens*. In the spectra we found two PSI-related DAS with 35–45 ps and 160–190 ps fluorescence lifetime and could assign them to PSI with a small (PSI-small) and large (PSI-large) antenna size. We assigned the fluorescence lifetime of 35–45 ps to PSI-small based on the isolated PSI-small fluorescence lifetime found in this work (Fig. 1D) and by (Liu et al. 2025). In other model organisms, PSI fluorescence lifetimes range from 20 to 25 ps in *Synechococcus elongatus* (cyanobacterium, (Bhatti et al. 2020), 65–75 ps in *Chlamydomonas reinhardtii* (green algae, (Ünlü et al. 2014), and 50–100 ps in *A. thaliana* (vascular plants, (P. R. Bos et al. 2023; Ihalainen et al. 2005; Wientjes et al. 2011). Even though PSI-small is structurally similar to PSI in vascular plants, its fluorescence lifetime is shorter (P. R. Bos et al. 2023; Gorski et al. 2022; Slavov et al. 2008; Ünlü et al. 2014; Wientjes and Croce 2012). PSI in vascular plants exhibits stronger red-shifted emission due to the presence of more “red forms”, Chl molecules that absorb and emit light above 700 nm, than PSI in *P. patens* (Croce et al. 2000; Gorski et al. 2022). These red forms cause an effective uphill transfer of excitation energy to the reaction centre, thereby slowing down the trapping process and lengthening the overall fluorescence lifetime in vascular plant PSI complexes. In contrast, the lower abundance of red forms in *P. patens* PSI leads to faster excitation trapping and therefore a shorter fluorescence lifetime.

By contrast, the PSI-large fluorescence lifetime of 160–190 ps is substantially larger than all the mentioned model species PSI lifetimes. The Lhcb9 dependent PSI-megacomplex contains roughly 1.5–1.7 times the Chl *a* content of PSI-LHCI, based on the isolated structure (Pinnola et al. 2018; Zhang et al. 2023). The fluorescence lifetime scales with the antenna size, assuming all Chls are iso-energetic, and the antenna are well-connected. For PSII with open reaction centres in plants this has experimentally been confirmed (Caffarri et al. 2011). Based on the antenna size and assumptions, the average fluorescence lifetime of PSI-large would be 60–70 ps compared to the 40 ps of PSI-small. The presence of red forms and the strength of the connection between the antenna and the core complex have a major influence on the fluorescence lifetime. Since the amount of red forms in PSI of *P. patens* is limited (Liu et al. 2025; SI Fig. 1), we suggest that the fluorescence lifetime component of 160–190 ps is related to a large PSI complex with loosely connected LHCII antenna. This LHCII might be part of the Lhcb9 dependent PSI-megacomplex. Previous work in *C. reinhardtii* shows that only a few energy transfer pathways are available from LHCII to the PSI core in the PSI-LHCI-LHCII complex, due to a large physical gap (Le Quiniou et al. 2015). This leads to a fluorescence decay component of ~ 100 ps after 475 nm excitation (Le Quiniou et al. 2015). Alternatively, LHCII present in the stroma lamellae could transfer energy to PSI without being part of a specific PSI megacomplex. Indeed, a fluorescence decay component of ~ 200 ps was resolved for LHCII to PSI excitation-energy transfer in *A. thaliana* stroma lamellae membranes from the LHCI lacking mutant (Schiphorst et al. 2022) and the levels of LHCII in the stroma lamellae of *P. patens* are enhanced compared to *A. thaliana* (Pinnola et al. 2015). Time-resolved fluorescence measurements on the PSI-megacomplex can reveal whether the 160–190 ps component arises from weakly connected LHCII within the complex or whether a contribution of nonspecifically associated LHCII is more likely. Unfortunately, we have not yet succeeded in isolating the PSI-megacomplex, and therefore this question remains to be answered in the future (Sun et al. 2023; Zhang et al. 2023).

### State transitions are active at low irradiance

State transitions balance the difference in absorption between PSI and PSII to optimize photosynthetic efficiency when incoming irradiance is limiting (Minagawa 2011; Ruban and Johnson 2009; Taylor et al. 2019). To induce state transitions in *P. patens* in the in vivo time-resolved fluorescence spectroscopy setup, various actinic light intensities were evaluated. In vascular plants, state transitions are induced with approximately 10–30% of the growth light intensity, because high light intensity inhibits the LHCII protein kinase Stn7 (Benson et al. 2015; Dietzel et al. 2011; Haldrup et al. 2001; Koskela et al. 2018; Nikkanen et al. 2019; Pribil et al. 2010; Rintamäki et al. 2000). We found that 3 µmol m^− 2^ s^− 1^ red actinic light combined with far-red light induces state 1, while the actinic light alone induces state 2, both without triggering significant qE (Figs. 3 and 4). Since *P. patens* was grown at 50 µmol m^− 2^ s^− 1^, 3 µmol m^− 2^ s^− 1^ is approximately 6% of the growth light intensity, thus in the light limiting regime. In nature, the species grows in moist open soil or in seasonally wet areas, so it could be exposed to a wide variety of light intensities. We observed a qT value of 10 +/- 3% on average at 3 µmol m^− 2^ s^− 1^ actinic light, falling within the reported range of 1–13% (Busch et al. 2013; Gerotto et al. 2022; Iwai et al. 2015; Pinnola and Bassi 2018). Therefore, the scale of state transitions is similar in *P. patens* and *A. thaliana* (qT value ranging from 8 to 13%), but they act at different light intensities (Benson et al. 2015; Damkjær et al. 2009; Sattari Vayghan et al. 2022; Wientjes et al. 2013a). State transitions optimize light harvesting most effectively in light regimes where qE does not dissipate part of the excitation energy. The quickly reversible qE type of NPQ in *P. patens* is activated at lower light intensities and show stronger quenching than in *A. thaliana* (Alboresi et al. 2010), and therefore it seems most likely that state transitions also act at lower light intensities.

### Time-resolved fluorescence suggests a role of PSI with long fluorescence lifetime in state transitions

We recorded in vivo time-resolved fluorescence spectra in state 1 (far-red light) and state 2 (3 µmol m^− 2^ s^− 1^ blue light). In WT, the contributions of the DAS of PSII (1.7–1.8 ns DAS) and PSI-large (150–180 ps DAS) differ for state 1 and state 2, while the PSI-small contribution is constant (Fig. 6A). This could be caused by activation of photosynthesis when the moss is exposed to blue light, opening an additional decay pathway for excited state Chls. Therefore, the same laser power could close the PSII reaction centres less effectively, causing an increase of the 150–180 ps component. This phenomenon should equally affect the spectra of the s*tn7* mutant. In *stn7* the PSII (1.6–1.8 ns) and the PSI-small (35–45 ps) contributions change slightly, although not significantly, upon a shift from state 1 to state 2 conditions, while the PSI-large (160–190 ps) contribution is constant. At low light intensity (3 µmol m^− 2^ s^− 1^), the decrease in the PSII 1.6–1.8 ns component in s*tn7* cannot be explained by qE quenching (see Fig. 3B) and therefore remains unexplained at present. As the increase in PSI-large fluorescence is only observed in the WT that may result from the state 1 to state 2 transition. Instead, the time-resolved fluorescence data indicate that the PSI-small complex does not substantially participate in state transitions, although we did observe a faint PSI-LHCI-LHCII band on the Blue-Native PAGE gel (Fig. 4). We did not resolve a PSI complex with large antenna size containing one or several LHCII’s, indicating that such complex is not stable in our β-DM detergent solubilisation condition.

Our data suggests that PSI-large mostly partakes in state transitions in *P. patens*, although the light treatment only induced minor amplitude variations. This is surprising, as the long PSI-large lifetime contributes approximately 8 times less to the PSI decay compared to PSI-small (based on the area under the DAS (Fig. 1B)). Further research is needed to evaluate if this is indeed the case and why LHCII would migrate to the small fraction of PSI complexes with a large antenna size instead of the more abundant PSI complexes with a small antenna size. Previous research reports that *P. patens* without Lhcb9 performs state transitions similar to the WT, suggesting that the PSI-megacomplex does not participate (Iwai et al. 2015; Pinnola and Bassi 2018). Nonetheless, these measurements are hard to compare to ours, because the used actinic light intensity was more intense (close to the growth light intensity) (Fig. 3B). Therefore, the fate of LHCII leaving PSII during a state transition in *P. patens* remains to be discussed. In green algae, it has been debated whether LHCII aggregates and quenches (Iwai et al. 2010) or associates with PSI (Nawrocki et al. 2016), while in vascular plants LHCII associates with PSI (Galka et al. 2012; Kouřil et al. 2005). We explore the hypothesis that LHCII leaving PSII could either aggregate and quench, or associate with PSI in *P. patens*. The expected fluorescence lifetime for aggregated and quenching LHCII is several hundreds of picoseconds (Ünlü et al. 2014), much longer than the lifetime we ascribe to PSI-large. So, we suggest the increase in contribution of the 165–180 ps component upon state transitions results from an increase in abundance of PSI-large instead of aggregated LHCII.

## Conclusion

In conclusion, we have observed the presence of two types of PSI-related fluorescence lifetimes in *P. patens*, namely 35–45 ps and 160–190 ps, pointing at a significant difference in their antenna size. These lifetimes are respectively assigned to PSI with a small antenna size (like in vascular plants, but without red forms) and PSI with a large, loosely connected antenna system. It remains unclear whether the PSI with large antenna size is the Lcb9 dependent PSI-megacomplex or whether it is PSI with non-specifically associated LHCII. State transitions are most effective in optimizing the light harvesting efficiency in low light conditions (i.e. 6% of the growth light intensity). In our experiments, mostly the PSI complex with large antenna size seems to be involved in state transitions rather than the PSI-small complex. Nonetheless the variations are minor and further research is needed to be conclusive.

## Supplementary Information

Below is the link to the electronic supplementary material.

Supplementary Material 1

## Data Availability

Data underpinning this publication is available on the data.4TU.nl data repository under doi 10.4121/50f65fe1-1aea-4722-9f35-cca4c84ac898.
