# Supplementary material for: State transitions in Physcomitrium patens studied with time-resolved fluorescence
Source: Photosynth Res. 2026 Mar 6;164(2):17. doi: 10.1007/s11120-026-01206-4 (PMC12963178; doi:10.1007/s11120-026-01206-4)
Supplement: Supplementary file 1 — Supplementary Material 1 [file 11120_2026_1206_MOESM1_ESM.docx]

**Supplementary Information**

***Physcomitrium patens*’ Photosystem I in action**

Dana Verhoeven^1^, Cleo Bagchus^1^, Lotte Jore^1^, Herbert van Amerongen^1^, Emilie Wientjes^1^*

**
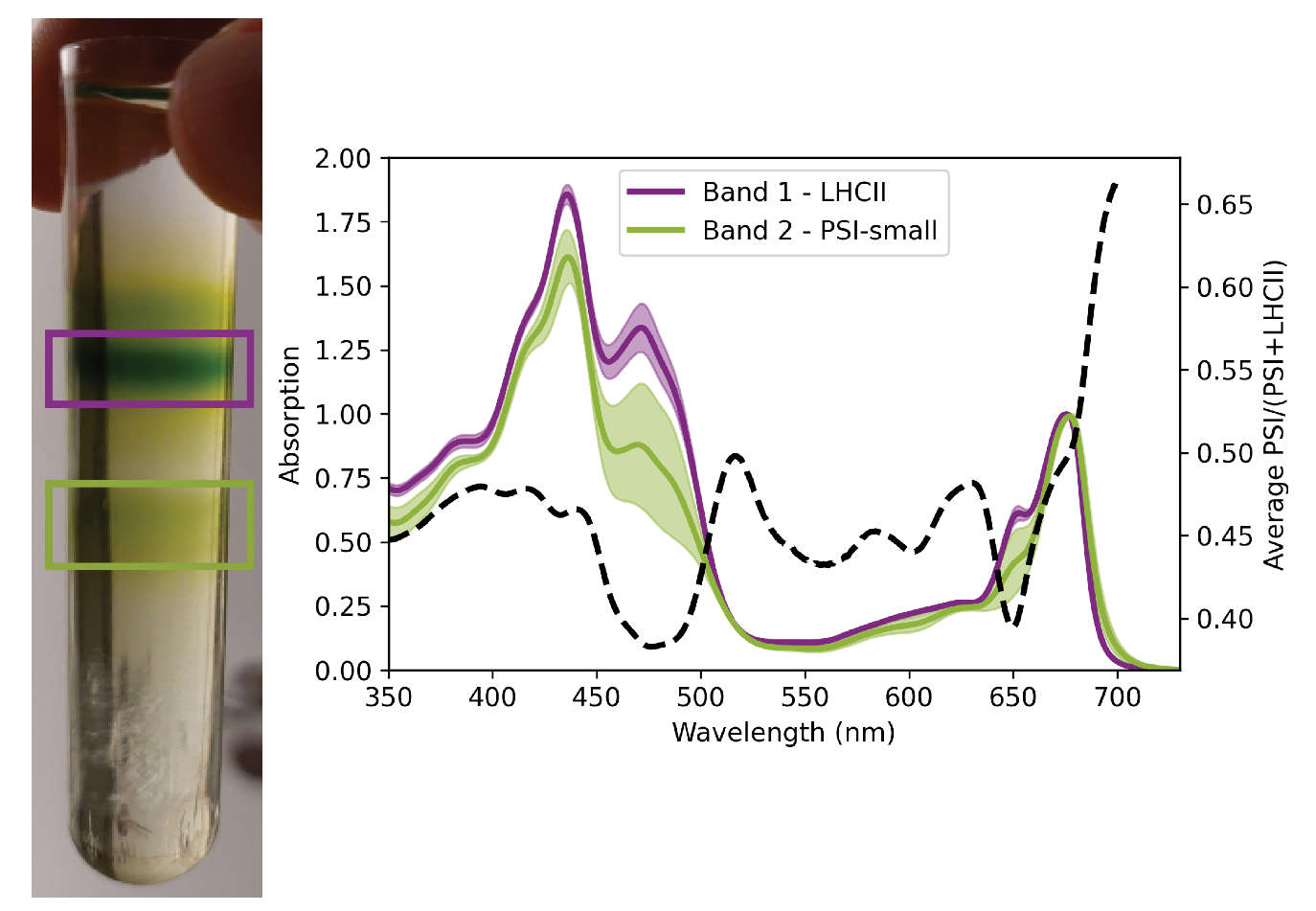
**^1^Laboratory of Biophysics, Wageningen University, 6708 WE Wageningen, The Netherlands.

SI Fig 1 Sucrose gradient on thylakoids dissolved with 1% β-DM and the absorption spectra of two selected fractions. The colour of the squares in the image is the same colour as the absorption spectrum of that fraction.

**
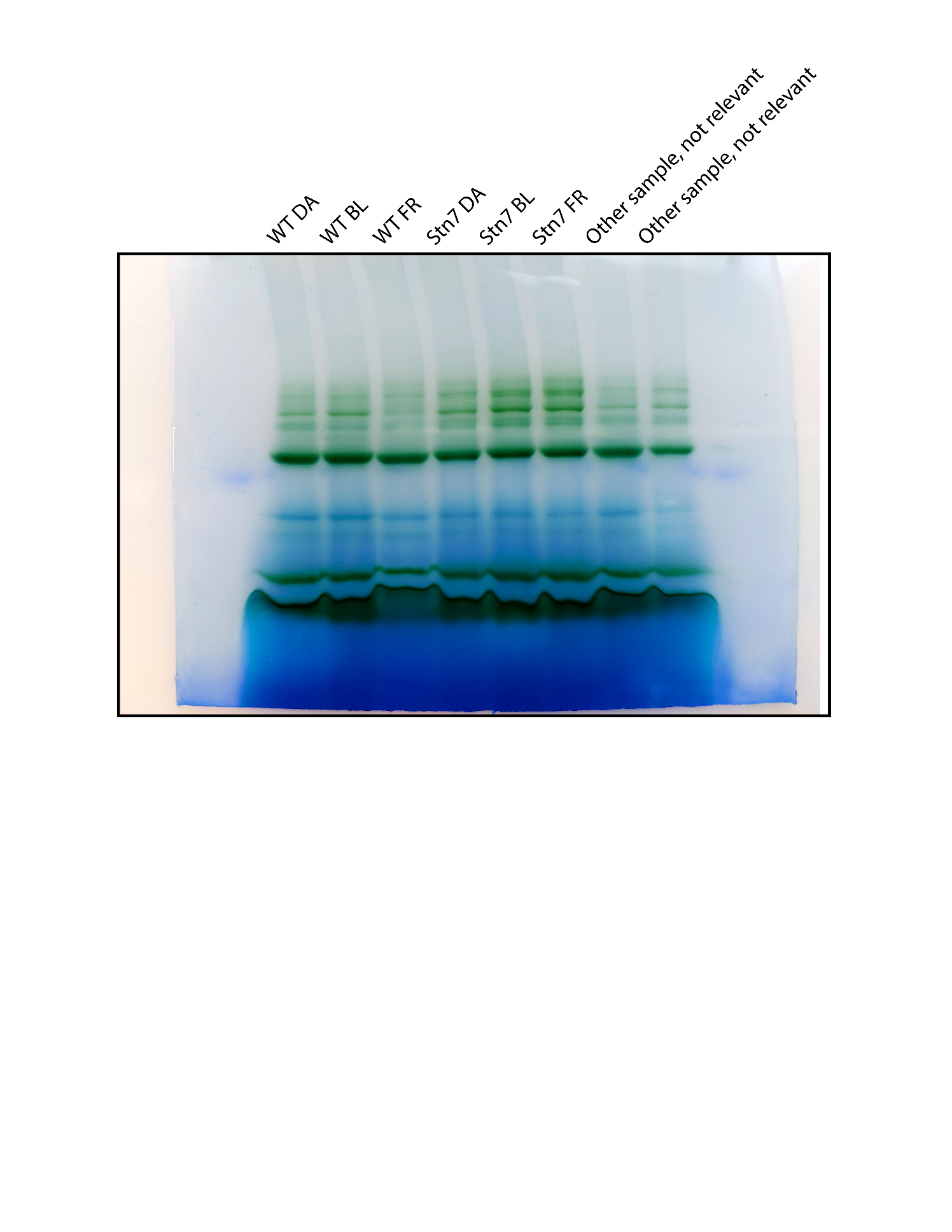
**

SI Fig 2 Blue-Native PAGE gel of WT and Stn7 thylakoids. The protonemata were dark adapted overnight and treated with either no light, 3 μmol m^-2^ s^-1^ blue light (BL) or 3 μmol m^-2^ s^-1^ far-red light (FR) for 30 min prior to the thylakoid isolation. The isolated thylakoids were dissolved with 1% β-DM.


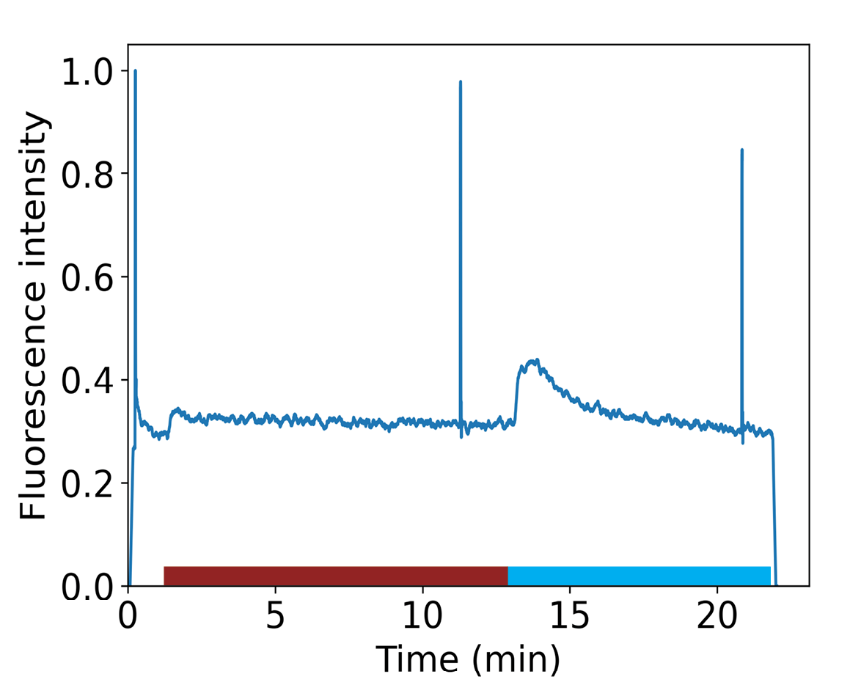


SI Fig 3 PAM state transition measurement in WT with the far-red and blue external lamp that are also used in the STREAK measurements in this work. The dark-red bar indicates when the far-red light (3 μmol m^-2^ s^-1^, λ_max_ = 706 nm) was on, and the blue bar indicates when the blue light (3 μmol m^-2^ s^-1^, λ_max_ = 488 nm) was on.
